# The Causation of Disease

**Published:** 1889-06-08

**Authors:** 


### The Causation of Disease.—III.*

We have still some observations to make on Dr. Campbell's "Causation of Disease." Having stated the proposition that disease depends on abnormal interaction between cell structure and cell environment, both internal and external, which includes also heredity, and having ably and learnedly supported this proposition, the author has something to say of the causation of special diseases. Some time ago Dr. Campbell came to the conclusion that malignant growths must be caused by parasites, and this view he now supports. The growth of a malignant tumour or cancer is most remarkable. A tissue which for many years has been healthy, suddenly becomes the reverse. The process is quite peculiar, for while the nutritive activity of the part is actually increased, the change is a retrogressive one--from healthy to diseased structure, or to a structure of inferior value in an evolutionary point of view. This, Dr. Campbell thinks, is strongly suggestive of a sudden and peculiar modification of cell environment; and he instances dark patches of grass as sometimes observed growing luxuriantly from some different soil, or environment, in that particular part of the ground. As regards the theory that malignant growths are developed from unused embryonic tissue or "remains," it is remarked that local irritation often determines the site of a cancer, and there is no reason why embryonic remains should reside specially in those positions more particularly exposed to irritation—the tongue, for example, or the alimentary canal. Yet it is in the tongue that cancer often developes, apparently from the irritation of a broken tooth; or in the lip, from the irritation of a pipe; or in the stomach, which is specially exposed to irritation from various kinds of food and drink. The following conclusions are then drawn: 1. That malignant disease is due to an abnormal cell environment. 2. That the abnormality consists in the presence of some noxious agent. 3. That the mal-environment consists of something not of the body. This cannot be irritation, for malignant tumours present independently of irritation. In the second place, malignant degeneration is continuous, and therefore is required a mal-environment which abides during the whole time. The form of mal-environment which meets all the peculiarities of the case is, the author thinks, one consisting of a living organism, and he takes it "as all but experimentally proved," that "under bacterial irritation the tissues are unable to keep at their normal level, and revert to a lower order of tissue." We are not, however, informed in what manner the bacteria enter the body, and in our opinion the diverse nature of cancerous growths is very much against the theory. In their order of similarity, if not of frequency, we have first scirrhus and encephaloid cancer, the appearances of which to the naked eye and touch are different, and also to a lesser extent under the microscrope. Scirrhus cancer is hard, while encephaloid is soft. On a section scirrhus shows a greyish circumference, a pinkish-yellow inner zone, and a white centre, while encephaloid is very much the same colour throughout. Under a high-power glass the circumference of scirrhus shows small round cells, the two other zones affording fibro-cellular stroma enclosing epitheloid cells and fatty degeneration. Encephaloid under the microscope does not certainly very much differ, but the cells are more numerous, generally larger, and there is less fibrous tissue. Secondly, epitheliomatous cancer shows layers of squamous epithelial cells. Thirdly, there is cylindrical epithelioma, which consists of irregular tubules lined with columnar epithelium. Fourthly, there is colloid cancer, in which fibrous bands are demonstrated containing jelly-like substance, in the centre of which is a granular mass, consisting of remains of altered cells. Now it follows that either there must be as many kinds of bacteria as there are varieties of cancer, or there must be some other agency in operation to produce such varieties of cancer. Dr. Campbell has not quite lost sight of all this, for he remarks, "There are probably several species of malignant bacteria." There is still, however, another objection. Fibrous and certain other tissues may constitute disease, and it would be quite as reasonable to theorise fibrous or fatty bacteria as cancerous. There is, moreover, the objection that cancer is a disease which is well known to be hereditary; therefore, if the exciting cause is bacteria, these microscopical agencies must be endowed with the sense of perception, at least to the extent of ascertaining in whom the hereditary taint, affording them a suitable nidus, resides.

We confess that we incline towards previous and less theoretical ideas of disease. We agree that disease is an abstraction or relation, and not an entity, having a special and independent existence, for, if the latter, it would appear equally in dead and living matter. In time, perhaps, we may be able to trace those early and minute changes constituting disease, and the causes which give them origin. But at present, even with the assistance of Dr. Campbell's book, we are not able to do so satisfactorily. When men's perceptive faculties are baffled, men dream, or, to use a milder term, theorise; when men's faculties compass their object, men enquire after cause. Hitherto we have been content to believe that whatever is capable of damaging the structure of any organ or tissue or of interfering with its functions may be a cause of disease. Under this view, the causes of disease have been classified into predisposing or remote, and exciting or determining, some of which, however, operate in both ways. Thus, there is no doubt that *age* has a most important influence on predisposition to disease, for there are various maladies more or less common, if not altogether peculiar, to certain periods of life. *Heredity*, again, must be regarded as a very powerful predisposing agency. *Intermarriage* is also a predisposing agency, the truth of which is often exemplified by the ravages of phthisis. *Sex*, again, predisposes to certain maladies. The influence of *temperament* cannot be ignored. *Differences of climate* may be both predisposing and an exciting cause of disease. *Locality*, again, influences in a similar manner. *Hygiene and sanitation*, or rather the neglect of hygiene and sanitation, will both predispose to and excite disease. A similar remark applies to *occupation*. *Previous disease* is often followed by similar or other maladies. *Mental and moral conditions* influence disease. *Extreme physical exertion* may cause disease. *Temperature*, *poisons*, *diet*, are all causes of disease. Of course all the above may be brought under Dr. Campbell's nomenclature of cell environment, external or internal, and they all no doubt lead to that abnormal interchange between cell structure and cell environment which Dr. Campbell regards as the ultimate essence of disease. But we may ask *cui bono?* We have long known that the contamination of the blood by the accumulation of waste product lower the vigour of the body generally, and renders it more ready to be affected by any disease to which it may be liable either by constitution or by acquirement. When any kind of mal-nutrition exists there must be premature degeneration and augmented waste, or, in other words, excessive generation of effete matter within the body. As an example, we may refer to the strong disposition to zymotic disease produced by intemperance. This is no doubt placing the matter in a coarser form than

* "An Exposition of the Ultimate Factors which induce it." By Harry Campbell, M.D., B.S., London, Member of the Royal College of Physicians; Assistant-Physician and Pathologist to the North-West London Hospital. H. K. Lewis, 136, Gower-street. 1889. (Continued from The Hospital of May 25, p. 115.)

that adopted by Dr. Campbell, but, at the same time, it is more practical. When natural phenomena are observed, or when, in fact, anything is observed, a tendency immediately developes to refer it to something previously known, to bring it within the range of acknowledged sequences, in brief, to define a cause. But the human system is so complex, every part standing in such intimate relation, the whole controlled by a vital principle of which we know nothing, that any attempt beyond mere generalisation has hitherto been failure. While scientists like Dr. Campbell work on, the majority must confine themselves to the at present more useful *role* of endeavouring to recognise the presence of disease, of estimating its progress and duration, of preventing its recurrence or appearance, and of curing it when it has occurred. The attainment of these *desiderata* is not much assisted by Dr. Campbell's theory that all disease depends on (1) peculiarity of cell or tissue structure; (2) peculiarity of cell environment; (3) peculiarity of both.

We have devoted as much time and space as we can afford to Dr. Campbell's "Causation of Disease." Not that we altogether agree with the arguments and conclusions of the author, or that we think our knowledge of the causation disease, and of the ultimate factors which induce it, has been very much increased by Dr. Campbell's work. But, on the other hand, it may be regarded as an honest attempt to do so. The volume extends over 360 pages, displays extensive research into all that has been written previously on the subjects on which it treats, and contains close critical argument and analysis which testify strongly to the mental power and scientific train of thought of the author. A notice of such a book in the limited space of a hebdomadal journal must necessarily be imperfect, and to a certain extent cursory. We can, therefore, only advise our readers to obtain Dr. Campbell's work and study it themselves. Although they may not be convinced, the time so devoted will not be ill-spent, especially if there is any previous acquaintance with the writings of the Duke of Argyll, Brown-Sequard, Darwin, Hilton Fagge, Galton, Hooker, Huxley, Knox, Paget, Sedgewick, Herbert Spencer, all of whom are drawn upon.

